# Obstructive Hydrocephalus Due to Aggressive Posterior Fossa Tumor Exhibiting Histological Characteristics of Pilocytic Astrocytoma in Two Adult Neurofibromatosis Type 1 (NF1) Cases

**DOI:** 10.7759/cureus.58697

**Published:** 2024-04-21

**Authors:** Shigeaki Nawa, Fumiharu Ohka, Kazuya Motomura, Kazuhito Takeuchi, Yuichi Nagata, Junya Yamaguchi, Ryuta Saito

**Affiliations:** 1 Department of Neurosurgery, Nagoya University Graduate School of Medicine, Nagoya, JPN

**Keywords:** brain tumors cns tumors, dna methylation, high-grade astrocytoma with piloid features, hydrocephalus, neurofibromatosis1 syndrome

## Abstract

Neurofibromatosis type 1 (NF1) is an autosomal dominant syndrome caused by germline alteration of the NF1gene. Among various NF1-related manifestations, obstructive hydrocephalus especially in adult NF1 cases is less frequently found. We report two adult NF1 cases exhibiting obstructive hydrocephalus due to an aggressive posterior fossa tumor exhibiting pathological characteristics of pilocytic astrocytoma as NF1-related manifestations. In these two cases, we performed endoscopic third ventriculostomy (ETV) and tumor biopsy as an initial treatment. The initial pathological diagnosis of the tumor is conventional pilocytic astrocytoma. After biopsy both cases revealed rapid tumor growth, therefore, we performed tumor removal, chemotherapy, and radiation therapy during an aggressive clinical course. However, both cases revealed dismal prognosis due to the progression of the tumor in spite of successful management of hydrocephalus by an initial ETV. DNA methylation analysis revealed that the tumor of one case matched high-grade astrocytoma with piloid features (HGAP). Most central nervous system tumors developed in NF1 are less aggressive such as pilocytic astrocytoma; however, recently a few studies revealed that HGAP, which has been a newly introduced malignant tumor in the World Health Organization Classification of Tumors of the Central Nervous System, 5th edition (WHO CNS 5), rarely develops in NF1 cases. These findings suggested that HGAP might be one of the important causes of obstructive hydrocephalus in adult NF1 cases.

## Introduction

Neurofibromatosis type 1 (NF1) is an autosomal dominant syndrome caused by germline alteration of the NF1 tumor suppressor gene. NF1 prevalence is approximately 1/3,000 worldwide [1, 2]. Among various clinical features, clinical diagnosis of NF1 is made by two or more features including the presence of more than six café-au-lait macules, skinfold freckling, Lisch nodules, characteristic lesions of the bone, optic pathway gliomas, neurofibromas of the skin or deep nerve, and a first-degree relative with NF1 [3]. Seizures and hydrocephalus are also reported to be one of the characteristic NF1-associated manifestations. NF1 cases sometimes develop obstructive hydrocephalus due to optic pathway glioma, other CNS tumors, NF1-associated tissue changes, and aqueductal stenosis. The incidence of hydrocephalus is reported to be 1-13% in pediatric NF1 cases [4,5]. By contrast, in adult NF1 cases, hydrocephalus is limited to approximately 1% [4]. Among CNS tumors, pilocytic astrocytoma (PA) is the most frequently found tumor in NF1 cases. Most of optic pathway gliomas, estimated to affect 15-20% of NF1 children, are PAs [6]. Most PA arising in the setting of NF1 syndrome exhibits a favorable prognosis; however, NF1-associated PA in adults is recognized as a more aggressive subtype [7]. We experienced two adult NF1 cases developing obstructive hydrocephalus due to an aggressive posterior fossa tumor exhibiting histological characteristics of PA. ETV is effective for the management of hydrocephalus, however, tumors exhibited refractory to various treatments including tumor removal, chemotherapy, and radiation therapy, leading to a dismal prognosis. Recently, in the World Health Organization Classification of Tumors of the Central Nervous System, 5th edition (WHO CNS 5), high-grade astrocytoma with piloid features (HGAP) has been introduced as a new entity [8]. Most frequently, HGAP originates in the posterior fossa. HGAP reveals similar pathological features with those of PA along with malignant histological features [9]. Although CDKN2A/B homozygous deletion and/or ATRX mutation is reported to be characteristic genetic alteration in HGAP, DNA methylation analysis is essential for the diagnosis of HGAP [10]. Although a few studies reported HGAP developed in NF1 cases, the clinical course of HGAP in NF1 cases has not been well elucidated [7,11]. In our two cases, one case revealed DNA methylation status matched with HGAP. This report aims to describe that for obstructive hydrocephalus developed in adult NF1 cases, malignant CNS tumors including HGAP might be detected.

## Case presentation

Case 1

**Figure 1:**
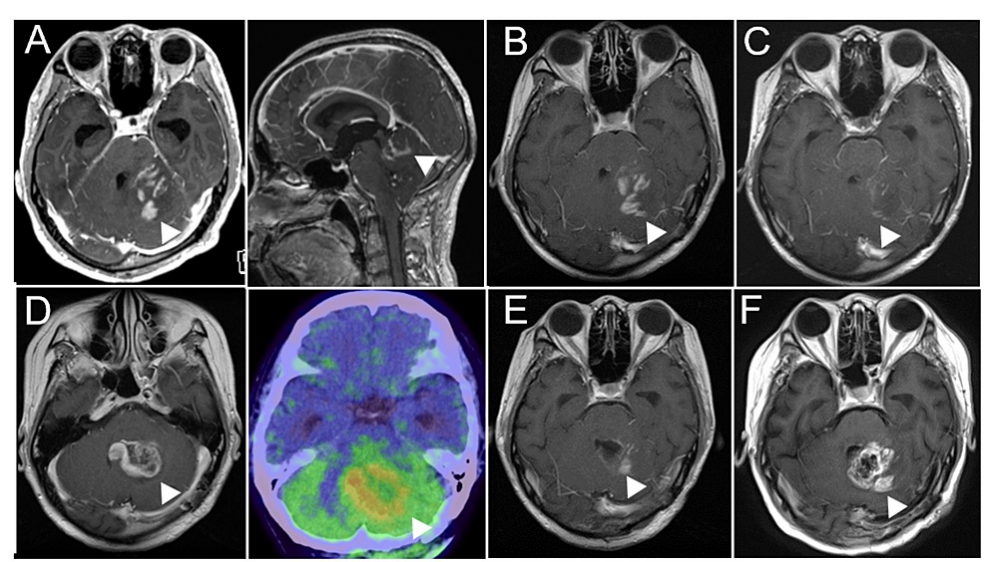
MRI and PET images during the clinical course of Case 1. (A) MRI contrast-enhanced T1-weighted axial (left) and sagittal (right) images at initial onset. (B) MRI image after chemotherapy with cisplatin and vincristine. (C) MRI image after radiation therapy (54Gy/30fr). (D) MRI image (left), and Methionine PET (right) at one month after radiation therapy. (E) MRI image after tumor removal. (F) MRI image after three months of tumor removal.

**Figure 2:**
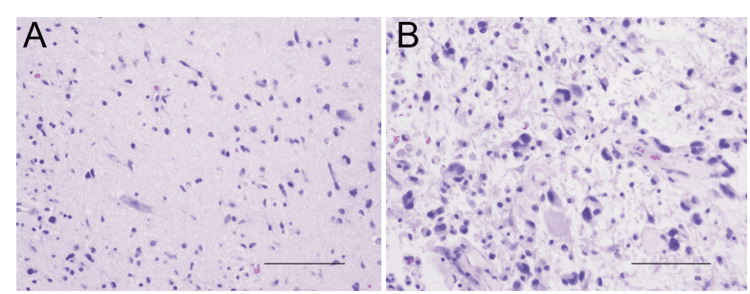
HE images of Case 1. (A) HE image of tumor obtained by biopsy. The scale bar indicates 100 μm. (B) HE image of tumor obtained by tumor removal. The scale bar indicates 100 μm. HE: Hematoxylin and eosin staining.

A 34-year-old male, whose father was diagnosed with NF1. There is no past medical history of NF1-associated manifestations. Since two months ago, he developed progressively worsening dizziness and gait disturbance along with diplopia and dysarthria. Head MRI images revealed a left cerebellar pontine tumor and obstructive hydrocephalus due to the tumor. Endoscopic third ventriculostomy (ETV) and tumor biopsy are performed (Figure 1A). The pathological diagnosis of the tumor specimen obtained by biopsy was pilocytic astrocytoma (PA) based on the findings of hematoxylin and eosin (HE) staining (Figure 2A).

After these procedures, dizziness, diplopia, and dysarthria were significantly improved. three courses of chemotherapy with cisplatin (20mg/m^2^; intravenous administration) and vincristine (1.4mg/m^2^; intravenous administration) were introduced without any obvious side effects, however, dizziness and dysarthria recurred two months later due to tumor regrowth (Figure 1B). Then, focal radiation therapy (54Gy/30fr) was performed. Just after completion of radiation therapy, once the mass effect was improved with radiation therapy, resulting in improvement of dysarthria (Figure 1C), one month later, tumor regrowth from the middle of the cerebellum to the left cerebellar hemisphere and pons was found. Methionine PET image revealed that the recurrent tumor exhibited a high accumulation of methionine (Figure 1D). We performed tumor removal except for a small amount of tumor adhering to the brainstem (Figure 1E). HE staining of the tumor revealed the proliferation of atypical glial cells with proliferation of vessels (Figure 2B). Immunohistochemistry (IHC) staining revealed that 5-10% of Ki-67 index. Postoperatively, despite the introduction of medication treatment using temozolomide (150 mg/m^2^; oral administration) and bevacizumab (10 mg/kg; intravenous administration), he passed away 16 months after the first diagnosis (Figure 1F). The Illumina Infinium Human Methylation EPIC v2.0 BeadChip array (Illumina, San Diego, CA, USA) was used for genome-wide methylation analysis. In total, 500 ng of DNA extracted from frozen specimens was used as the input material. The output data (IDAT files) were checked for general quality as indicated by the manufacturer. A molecular classification algorithm and copy number analysis from the German Cancer Center (DKFZ classifier, https://www.molecularneuropathology.org/mnp) was performed [12]. DNA methylation-based classification using the DKFZ classifier revealed that the tumor of Case 1 was matched to the HGAP subtype with a high calibrated score (0.96). A log2 ratio of −0.415 was used as the cut-off for homozygous loss. The tumor of Case 1 revealed CDKN2A/B homozygous deletion.

Case 2

**Figure 3:**
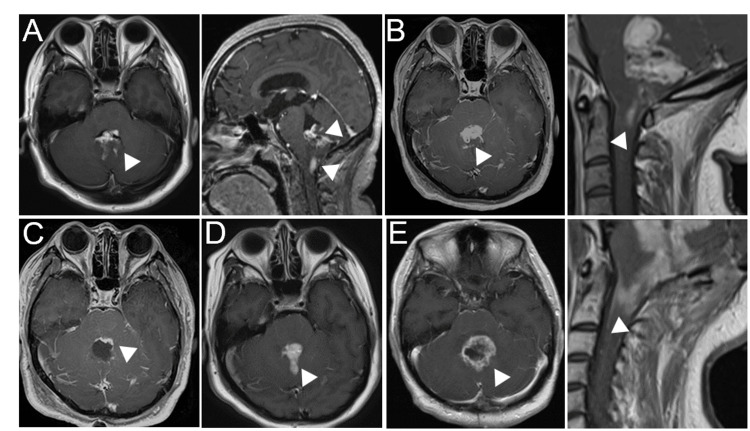
MRI images during the clinical course of Case 2. (A) MRI contrast-enhanced T1-weighted images (left; head axial image and right; head and cervical sagittal image) at initial onset. (B) MRI images (left; head axial image and right; cervical sagittal image) at regrowth of the tumor. (C) MRI image after tumor removal. (D) MRI image after two months of tumor removal. (E) MRI images (left; head axial image and right; cervical sagittal image) after radiation therapy.

**Figure 4:**
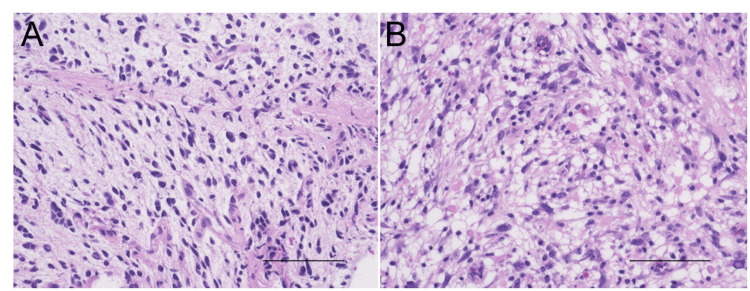
HE images of Case 2. (A) HE image of tumor obtained by biopsy. The scale bar indicates 100 μm. (B) HE image of tumor obtained by tumor removal. The scale bar indicates 100 μm. HE: Hematoxylin and eosin staining.

A 37-year-old woman, who was diagnosed with NF1 in childhood. She has no family history related to NF1 in the parents. Since two months ago, she developed numbness in both lower extremities, dizziness, and left-sided visual disturbance. Head MRI images revealed a tumor extending from the cerebellar vermis to the floor of the fourth ventricle, dissemination to the spinal cord, and obstructive hydrocephalus (Figure 3A). We performed ETV and tumor biopsy. The pathological diagnosis of the tumor was low-grade astrocytoma, consistent with pilocytic astrocytoma (PA; Figure 4A).

Three months later, the tumor revealed significant growth (Figure 3B). Therefore, subtotal removal of the tumor was performed five months after initial diagnosis. A small amount of tumor adhering to the brainstem or the floor of the fourth ventricle was left (Figure 3C). The obtained tumor specimen revealed atypical glial cells with Rosenthal fiber, consistent with those of pilocytic astrocytoma (Figure 4B). IHC revealed that 5-10% of the Ki-67 index. After two months of tumor removal, the residual tumor revealed regrowth immediately. Focal radiation therapy (60Gy/30fr) was performed without any obvious side effect, however, after completion of radiation therapy, the tumor revealed regrowth immediately, resulting in worsening of dysarthria and dysphagia (Figure 3D). She died 12 months after the first diagnosis (Figure 3E). Using the same method, we analyzed DNA methylation status and copy number alterations. DKFZ classifier revealed that the tumor of Case 2 did not match, however, presented the highest calibrated score (0.71) for the HGAP subtype. The tumor of Case 2 also revealed CDKN2A/Bhomozygous deletion by FoundationOne CDx testing.

## Discussion

We experienced two adult NF1 cases exhibiting obstructive hydrocephalus due to aggressive posterior fossa tumor as NF1-related manifestations. While NF1-related tumors are often benign type, these cases revealed dismal prognoses due to the progression of the tumor in spite of successful management of hydrocephalus. Although Case 2 did not match HGAP, DNA methylation analysis revealed that the tumors of both cases revealed high calibrated scores for HGAP (Case 1; 0.96 and Case 2; 0.71). PA is the most commonly found in children, however, a subtype of this tumor reveals an aggressive clinical course through malignant transformation, recurrence, or dissemination. PA developed in NF1 cases exhibits a more aggressive clinical course than those developed spontaneously [7]. For the diagnosis of HGAP, DNA methylation analysis is essential along with pathological examination. HGAP often exhibits gene mutations in NF1, BRAF, or FGFR1 in combination with CDKN2A/B homozygous deletion and/or ATRX mutations or loss [10]. Although HGAP rarely shows malignant histopathology, the prognosis of most cases is unfavorable like malignant diffuse glioma [9]. In recent years, with the development of comprehensive genetic profiling methods, the number of reports of HGAP developed in NF1 cases has increased. Immediate DNA methylation profiling during the clinical course is quite important for the prediction of the prognosis in PA developed in NF1 cases, especially for the case with aggressive clinical course as did for our cases. In our two cases, dissemination is found from the initial onset. Dissemination findings on the initial onset might be one of the important findings of an aggressive phenotype in PA developed in NF1. Therapy for distinct approaches is engaged for major subclasses in HGAP, such as MAPK kinase inhibitors (MEKi), PI3K/mTOR inhibitors, cyclin-dependent kinase inhibitors, and ATR inhibitors. Concurrent radiotherapy might be important for HGAP treatment [13]. However, a standard treatment strategy for HGAP has not been established yet.

The frequency of hydrocephalus has been reported to be higher in pediatric NF1 cases than those in adult NF1 cases. Hydrocephalus in adult NF1 is a complication with a low probability of approximately 1%, and in a study that focused only on NF1 and compiled complications, NF1-related hydrocephalus was extremely rare, accounting for only 1 case out of 138 cases [4]. Treatment often involves a combination of treatments of the cerebrospinal fluid pathway, tumor excision, chemotherapy, and observation. However, NF1-related hydrocephalus is often refractory, and a relatively high rate of failure has been described in hydrocephalus control [14, 15]. In these two cases, we could successfully manage hydrocephalus by ETV for initial treatment, however, we could not inhibit tumor growth. For treatment for hydrocephalus due to posterior fossa tumor in adult NF1 cases, immediate DNA methylation analysis of tumor tissue obtained by tumor biopsy and ETV might be quite important for predicting aggressive clinical course.

## Conclusions

We experienced two adult neurofibromatosis type 1 (NF1) cases exhibiting hydrocephalus due to an aggressive posterior fossa tumor. The initial pathological diagnosis of these two cases was conventional pilocytic astrocytoma. DNA methylation analyses revealed that the tumor of Case 1 matched HGAP (high-grade astrocytoma with piloid features; 0.96). Case 2 did not match HGAP, however, exhibited a high calibrated score for HGAP (0.71). Both cases revealed dismal prognosis due to not hydrocephalus but tumor progression in spite of variable treatment. For treatment for adult NF1 cases exhibiting hydrocephalus due to posterior fossa tumor, immediate DNA methylation analysis of tumor tissue obtained by tumor biopsy and endoscopic third ventriculostomy might be quite important for predicting aggressive clinical course.

## References

[1] Gutmann DH, Ferner RE, Listernick RH, Korf BR, Wolters PL, Johnson KJ (2017). Neurofibromatosis type 1. Nat Rev Dis Primers.

[2] Uusitalo E, Leppävirta J, Koffert A (2015). Incidence and mortality of neurofibromatosis: a total population study in Finland. J Invest Dermatol.

[3] (1988). Neurofibromatosis. Conference statement. National Institutes of Health Consensus Development Conference. Arch Neurol.

[4] Créange A, Zeller J, Rostaing-Rigattieri S, Brugières P, Degos JD, Revuz J, Wolkenstein P (1999). Neurological complications of neurofibromatosis type 1 in adulthood. Brain.

[5] Dinçer A, Yener U, Özek MM (2011). Hydrocephalus in patients with neurofibromatosis type 1: MR imaging findings and the outcome of endoscopic third ventriculostomy. AJNR Am J Neuroradiol.

[6] Cassina M, Frizziero L, Opocher E (2019). Optic pathway glioma in type 1 neurofibromatosis: review of its pathogenesis, diagnostic assessment, and treatment recommendations. Cancers (Basel).

[7] Romo CG, Piotrowski AF, Campian JL (2023). Clinical, histological, and molecular features of gliomas in adults with neurofibromatosis type 1. Neuro Oncol.

[8] Louis DN, Perry A, Wesseling P (2021). The 2021 WHO classification of tumors of the central nervous system: a summary. Neuro Oncol.

[9] Bender K, Perez E, Chirica M (2021). High-grade astrocytoma with piloid features (HGAP): the Charité experience with a new central nervous system tumor entity. J Neurooncol.

[10] Reinhardt A, Stichel D, Schrimpf D (2018). Anaplastic astrocytoma with piloid features, a novel molecular class of IDH wildtype glioma with recurrent MAPK pathway, CDKN2A/B and ATRX alterations. Acta Neuropathol.

[11] Cimino PJ, Ketchum C, Turakulov R (2023). Expanded analysis of high-grade astrocytoma with piloid features identifies an epigenetically and clinically distinct subtype associated with neurofibromatosis type 1. Acta Neuropathol.

[12] Capper D, Jones DT, Sill M (2018). DNA methylation-based classification of central nervous system tumours. Nature.

[13] Raghu AL, Chen JA, Valdes PA (2022). Cerebellar high-grade glioma: a translationally oriented review of the literature. Cancers (Basel).

[14] Roth J, Ber R, Wisoff JH (2017). Endoscopic third ventriculostomy in patients with neurofibromatosis type 1: a multicenter international experience. World Neurosurg.

[15] Roth J, Ber R, Constantini S (2019). Neurofibromatosis type 1-related hydrocephalus: treatment options and considerations. World Neurosurg.

